# Recurrent Anthrax Outbreaks in Humans, Livestock, and Wildlife in the Same Locality, Kenya, 2014–2017

**DOI:** 10.4269/ajtmh.18-0224

**Published:** 2018-08-13

**Authors:** Mathew Muturi, John Gachohi, Athman Mwatondo, Isaac Lekolool, Francis Gakuya, Alice Bett, Eric Osoro, Austine Bitek, S. Mwangi Thumbi, Peninah Munyua, Harry Oyas, Obadiah N. Njagi, Bernard Bett, M. Kariuki Njenga

**Affiliations:** 1 Kenya Zoonotic Disease Unit, Nairobi, Kenya;; 2 Washington State University Global Health Program-Kenya, Washington State University, Pullman, Washington;; 3 Kenya Wildlife Services, Nairobi, Kenya;; 4 Food and Agriculture Organization of the United Nations, Nairobi, Kenya;; 5 Division of Global Health Protection, United States Centers for Disease Control and Prevention, Nairobi, Kenya;; 6 Kenya Directorate of Veterinary Services, Nairobi, Kenya;; 7 International Livestock Research Institute, Nairobi, Kenya

## Abstract

Epidemiologic data indicate a global distribution of anthrax outbreaks associated with certain ecosystems that promote survival and viability of *Bacillus anthracis* spores. Here, we characterized three anthrax outbreaks involving humans, livestock, and wildlife that occurred in the same locality in Kenya between 2014 and 2017. Clinical and epidemiologic data on the outbreaks were collected using active case finding and review of human, livestock, and wildlife health records. Information on temporal and spatial distribution of prior outbreaks in the area was collected using participatory epidemiology. The 2014–2017 outbreaks in Nakuru West subcounty affected 15 of 71 people who had contact with infected cattle (attack rate = 21.1%), including seven with gastrointestinal, six with cutaneous, and two with oropharyngeal forms of the disease. Two (13.3%) gastrointestinal human anthrax cases died. No human cases were associated with infected wildlife. Of the 54 cattle owned in 11 households affected, 20 died (attack rate = 37%). The 2015 outbreak resulted in death of 10.5% of the affected herbivorous wildlife at Lake Nakuru National Park, including 745 of 4,500 African buffaloes (species-specific mortality rate = 17%) and three of 18 endangered white rhinos (species-specific mortality rate = 16%). The species mortality rate ranged from 1% to 5% for the other affected wildlife species. Participatory epidemiology identified prior outbreaks between 1973 and 2011 in the same area. The frequency and severity of outbreaks in this area suggests that it is an anthrax hotspot ideal for investigating risk factors associated with long-term survival of anthrax spores and outbreak occurrence.

## INTRODUCTION

Anthrax, a bacterial zoonosis of global health security and public health importance, is primarily a disease of domestic and wild herbivores transmitted through ingestion of bacterial spores from soil and/or vegetation.^1,2^ Natural human infection occurs through contact with infected animal carcasses or contaminated animal products.^2,3^ Human anthrax is classified into three forms depending on the route of transmission; cutaneous (the most common globally accounting for up to 95% of all anthrax cases), inhalational, and ingestion form. The ingestion form of anthrax is further classified to an oropharyngeal or gastrointestinal form depending on site of infection and clinical manifestation.^2,4^ The disease has a global distribution but incidence in livestock and humans varies with local ecology, implementation of control strategies, and sociocultural practices that determine spillover from animals to humans.^5^ Although most developed countries report few sporadic cases in livestock and humans, the disease is still enzootic in parts of Africa, the Middle East, and Central Asia.^5–10^ Similarly, outbreaks of anthrax in wildlife have also been reported in diverse ecosystems globally: North America, Europe, tropical rain forests, and sub-Saharan Africa.^11–17^

The geographic distribution of anthrax is associated with certain ecological factors that promote viability and survival of *Bacillus anthracis* spores. Soil properties (type, alkalinity, nutrient composition, and moisture content), ambient temperature, and humidity are thought to be key ecological drivers.^2,7,17,18^ In some ecosystems, outbreaks occur late in the hot-dry season, whereas in others, outbreaks are associated with the end of heavy rains, suggesting that weather extremes may be an important trigger of outbreaks.^7,19–20^ Ecological drivers may, however, vary across ecosystems.^21^

Although anthrax remains a disease of public health importance in sub-Saharan Africa, weak surveillance systems result in an underestimation of the morbidity, mortality, and socioeconomic impact of the disease. In Kenya, anthrax is the highest ranked priority zoonotic disease, based on a systematic analysis of burden, socioeconomic impact, epidemic potential, and severity of disease.^22^ According to Kenya medical and veterinary records, an average of 10 anthrax outbreaks occur annually at the human–animal interface, likely an underestimate of the true burden of disease. In 2007, a national seroprevalence survey in humans reported *B. anthracis* seropositivity of 11.3%, with some regions of the country reporting up to 28% seropositivity.^23^ Animal and human outbreak records suggest that certain regions of the Kenya, including Murang’a, Nakuru, and Bomet counties experience recurrent anthrax outbreaks involving livestock and humans, suggesting that certain ecological areas are permissive to persistence of *B. anthracis* spores, with livestock and wildlife in the areas at risk of infection. Outbreaks in Kenyan wildlife have also been reported, but the complexity and challenges of disease surveillance in wildlife mean that many more cases are missed.^6,20,24^

Here, we describe three anthrax outbreaks involving humans, livestock, and wildlife that occurred in 2014, 2015, and 2017 in Nakuru West subcounty near Lake Nakuru National Park (LNNP) in Nakuru County, Kenya. In addition, we used participatory epidemiology (PE) techniques to identify and map historical anthrax outbreaks in the area dating back to 1970.

## METHODS

### Outbreak site.

**Figure 1.**
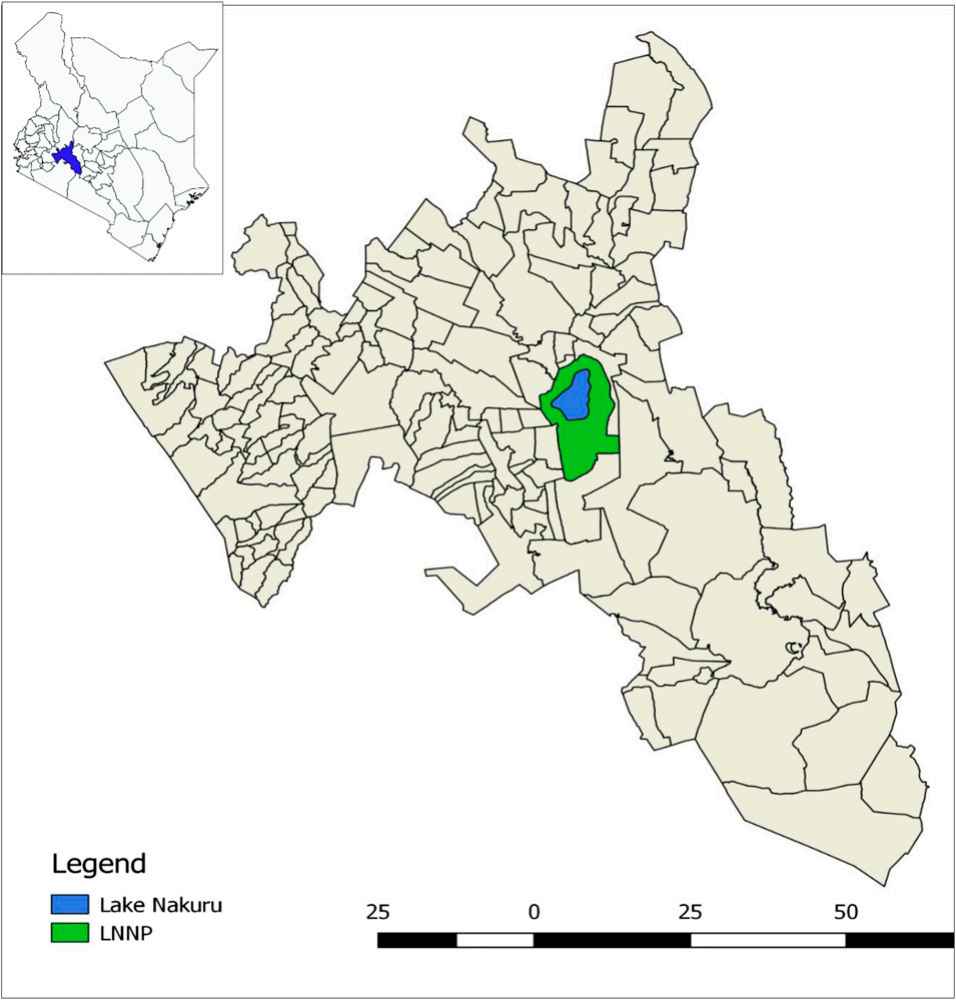
Map of Kenya showing Nakuru County in blue and the enlarged map of that of Nakuru County showing the location of Lake Nakuru and the surrounding Lake Nakuru National Park (LNNP) (green). This figure appears in color at www.ajtmh.org.

Although in Kenya, a single case of animal or human anthrax is considered an outbreak, most of the reported anthrax outbreaks involved at least one livestock and one human being. The three outbreaks described here occurred in Nakuru West subcounty, which lies on the eastern side of the Great Rift Valley in Kenya. Outbreaks in humans and livestock affected Soimet and Elementaita villages, whereas outbreaks in wildlife were confined to LNNP, which is adjacent to the two villages. The LNNP covers 188 km^2^ around Lake Nakuru, an alkaline lake on the floor of the Great Rift Valley (Figure 1). The outbreak area has an average annual rainfall of 1,000 mm, most of it falling in two seasons: long rains in April–August and short rains between November and December.^25^ The park is rich in wildlife diversity with 70 mammalian species within its ecosystem. In addition, the park has the highest concentration of the endangered black rhino species in Kenya.^26^

### Outbreak investigations.

A multidisciplinary team of medical and veterinary experts responded to each of the three outbreaks. The teams used quantitative and qualitative approaches to characterize the outbreaks. In the quantitative approach, the team conducted active case searching using World Health Organization (WHO) and World Organization for Animal Health (Office International des Epizooties [OIE]) anthrax case definitions for humans and animals, respectively.^2^ Using key informant interviews and snowballing technique, affected households and herds were identified. The total number of persons in households with at least one livestock case and total number of people found to have had contact with a suspected livestock case or its products through active case searching constituted the population at risk. The numbers of animals at risk were the total number of livestock (cattle, sheep, and goats) in herds/households reporting at least one suspect case of livestock anthrax. A suspect human case was a clinically compatible case of illness of any of the three forms during the outbreak period; a probable case was a suspect case epidemiologically linked to a confirmed environmental exposure, whereas a confirmed case was a clinically compatible case with isolation of *B. anthracis*. A suspect animal anthrax case was defined as sudden death with at least one of the following symptoms: oozing of unclotted blood from natural orifices or rapid bloating of the carcass or lack of rigor mortis. A confirmed animal anthrax case was defined as a suspect case in which rod-shaped spores were identified from a blood smear by microscopy as per OIE procedures.^27^ A semistructured standard questionnaire was used to collect demographic and risk factor information for human and animal cases.

Participatory epidemiology techniques were used in Soimet village that was affected by the 2014 and 2017 outbreaks. During the 2017 outbreak, two focus group discussions with eight participants each—one for men and the other for women—were held with livestock-owning residents of Soimet village. Two participatory approaches were used during the focus group interviews—semistructured interviews and participatory mapping. For mapping, participants identified locations and the timeline of anthrax outbreaks before 2017 on the map. All places identified to have had outbreaks were visited and georeferenced as shown in Figure 3. Other information collected during the interviews included practices that influence risk of infection in the humans and livestock. During the investigation of outbreaks in wildlife, sites of wildlife cases were georeferenced and information was collected on species, sex, and age of the affected animals.

### Outbreak confirmation.

During each outbreak, blood smears were taken from up to five randomly selected cattle carcasses and presence of classical gram-positive capsulated *B. anthracis* rods confirmed by microscopy at the Nakuru Regional Veterinary Investigation Laboratory. For the 2015 outbreak in wildlife, blood smears from seven randomly selected African buffalo (*Syncerus caffer*) and three rhino (*Ceratotherium simum*) carcasses were laboratory confirmed for the bacterium.

## RESULTS

### Outbreaks in livestock and humans.

**Figure 2.**
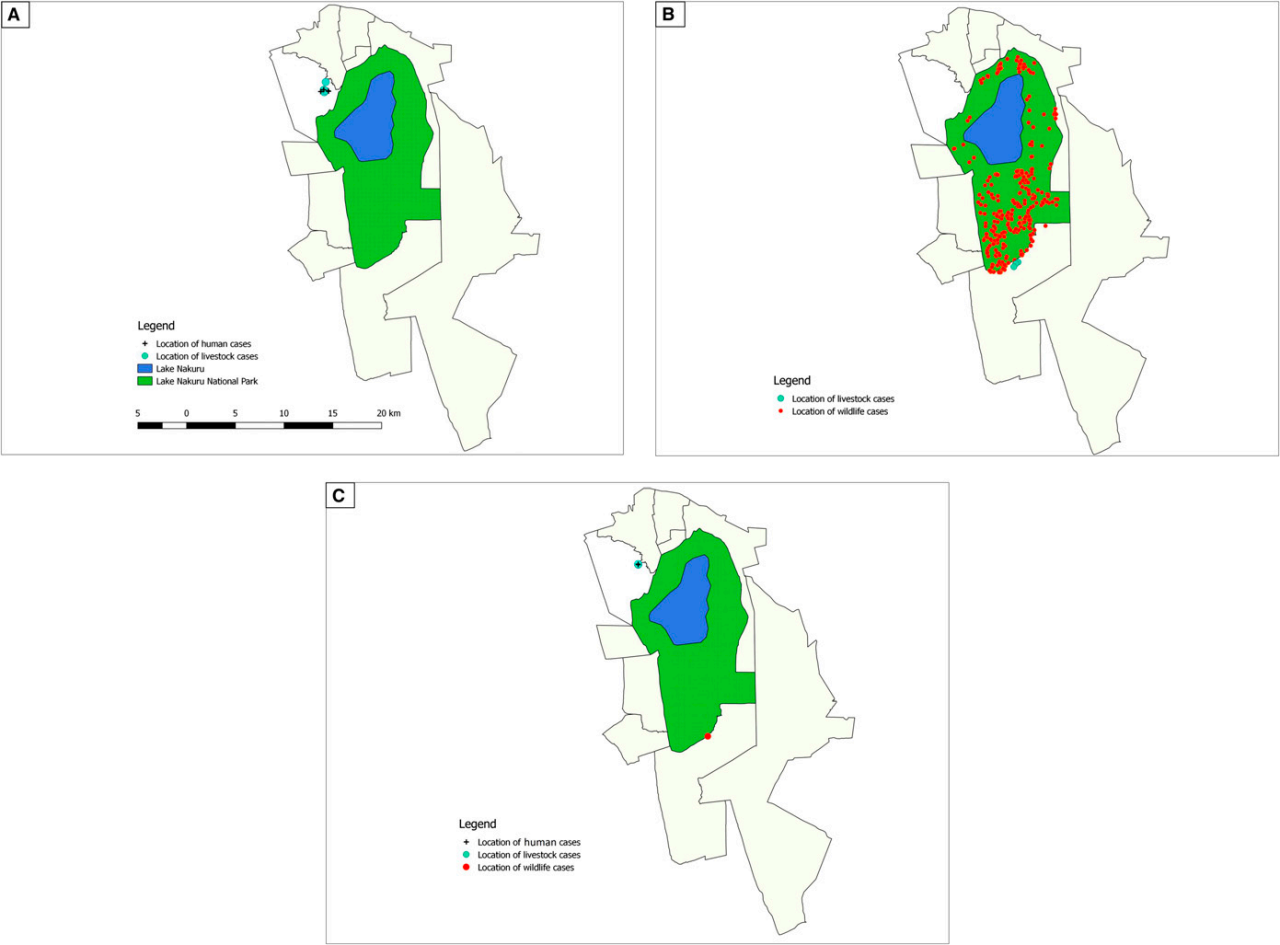
Maps of Nakuru West subcounty in Kenya showing spatial distribution of anthrax cases during the disease outbreaks in 2014 (**A**), 2015 (**B**), and 2017 (**C**). The location of Lake Nakuru is shown in blue, whereas the surrounding Lake Nakuru National Park is illustrated by the green. Locations of human cases are marked by +, livestock cases by blue circles, and wildlife cases by red circles in each map. This figure appears in color at www.ajtmh.org.

The anthrax outbreaks of 2014 and 2017 affected humans, livestock, and wildlife, whereas the 2015 outbreak affected livestock and wildlife only. Figure 2 is the area map showing proximity of the locations where human, livestock (cattle), and wildlife cases were reported during the 2014, 2015, and 2017 outbreaks.

The human outbreaks of 2014 and 2017 resulted in 15 probable cases (Table 1) with attack rates of 15% and 29%, respectively (Table 1). The number of people at risk (*N* = 71) was the total number of people in the households reporting a livestock case. The mean age of the cases was 27 years (standard deviation ± 15.4) with 87% (13/15) of the probable cases being male. The majority of the human cases presented with the gastrointestinal form (*N* = 7; 46. 7%) followed by the cutaneous (*N* = 6; 40.0%) and oropharyngeal (*N* = 2; 13.3%) forms. Two gastrointestinal cases died 2 days after exposure, giving a case fatality rate of 13.3% (2/15). Black eschars were the main clinical presentation of the cutaneous cases, whereas acute diarrhea and vomiting were the clinical features of the gastrointestinal form of the disease. Painful swallowing, swelling of the oropharyngeal region, and hemoptysis (coughing of blood) were the symptoms reported by both oropharyngeal cases. The mean incubation period was 4 days for the cutaneous, 1 day for gastrointestinal, and 6 days for the oropharyngeal forms. All human cases were associated with butchering dead cattle and/or consuming meat from such carcasses during the outbreak period.

**Table 1:** Species affected by the anthrax outbreaks of 2014, 2015, and 2017 in Nakuru West Subcounty, Kenya

Year	2014	2015	2017
Species affected (cases)	Humans (*N* = 6)	Human (*N* = 0)	Humans (*N* = 9)
	Cattle (*N* = 8)	Cattle (*N* = 10)	Cattle (*N* = 2)
	Wildlife (*N* = 0)	Wildlife (*N* = 766)*	Wildlife (*N* = 2)*
Location			
Human, cattle	Soimet village	Elementaita village	Soimet village
Wildlife	LNNP	LNNP	LNNP
Time of year	February–March	July–August	June–July

LNNP = Lake Nakuru National Park.

* Wildlife species and numbers affected in the 2015 and 2017 outbreaks are shown in Table 2.

Cattle were infected in all three outbreaks, whereas sheep and goats, which were also present in the same households, were not affected. In both villages, all affected livestock were nonindigenous adult cattle kept in households within a 500-m radius. A total of 54 cattle in 11 households were at risk, resulting in 20 deaths and an attack rate of 42% (8/19) in 2014, 37% (10/27) in 2015, and 25% (2/8) in 2017.

#### Anthrax in wildlife.

The 2015 outbreak at the LNNP resulted in mortality of 766 wild herbivores between July and August (Table 2). The highest species-specific mortality rate was recorded in African buffaloes (*S. caffer*) and white rhino (*C. simum*) as shown in Table 2. The 2017 outbreak killed two African buffaloes in the southern side of the park (attack rate = < 1%). Majority (*N* = 238; 58%) of affected wildlife were males, and almost all were adults (*N* = 443; 98%) in good body condition. The animals died suddenly without showing any clinical presentation. The main postmortem presentation was exudation of watery blood from all orifices and bloating of carcasses.

**Table 2:** Species and mortality rates of wildlife affected by anthrax outbreaks of 2015 (July–August) and July 2017 at LNNP, Nakuru West Subcounty*

Species	Number dead	Number at risk	Species specific mortality rates (%)	Overall mortality rate (%)
2015 outbreak				
Buffaloes	745	4,500	17	97
Black rhinoceros	5	60	8	< 1
White rhinoceros	3	18	16	< 1
Elands	4	75	5	< 1
Impalas	4	1,800	< 1	< 1
Thompsons gazelles	2	450	< 1	< 1
Rothschild giraffes	1	90	< 1	< 1
Warthogs	1	300	< 1	< 1
Waterbucks	1	Unknown	–	< 1
Total	766	7,293	–	10.5
2017 outbreak				
Buffaloes	2	4,000	< 1%	–

LNNP = Lake Nakuru National Park.

* No wildlife at LNNP were affected by the 2014 outbreak. Source: Kenya Wildlife Service.

#### Identification of past outbreaks.

**Figure 3.**
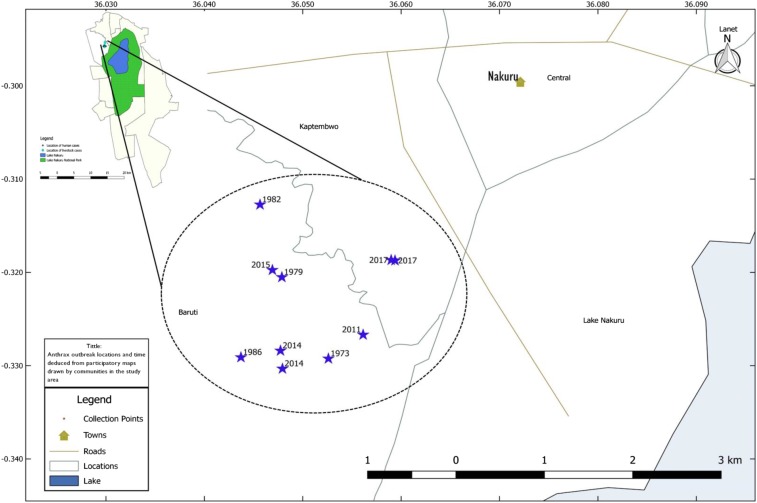
Map of Baruti sub-location showing frequency and spatial mapping of anthrax outbreaks between 1973 and 2017 (shown in blue stars) using participatory epidemiology. Soimet village is located in Baruti sub-location within Nakuru West sub-location (shown in insert). Lake Nakuru shown in blue, whereas the surrounding Lake Nakuru National Park is illustrated in green. This figure appears in color at www.ajtmh.org.

We used PE techniques to characterize current and past outbreaks in Soimet village in Baruti sub-location, which was affected in the 2014 and 2017 outbreaks. The livestock species kept in the village included cattle, sheep, and goats. The livestock are reared in a semi-intensive production system where cattle are fed with pasture from the individual pieces of land or outsourced feed from the nearby Nakuru town or from an open field near the LNNP. Both focus group discussions (FDG) were familiar with anthrax, which has a local name “*burasta*.” The FGDs reported prior anthrax outbreaks in the area in 1973, 1979, 1982, 1986, 2011, 2014, 2015, and 2017. Figure 3 shows the locations of all anthrax outbreaks in the area from 1973 to 2017.

Livestock keepers associated anthrax with convulsions, difficult breathing, sudden death, grunting, bloating immediately after death, failure of blood to clot, and splenomegaly on opening the carcass. Several sociocultural practices were identified as potential pathways for transmission of spores to humans and contamination of the environment with spores. Some villagers butcher and consume the meat from infected carcasses. Others butcher, not for consumption but to feed their dogs with it. Still, in the absence of consumption by either humans or dogs, carcasses are normally opened before burial as dictated by local culture. Finally, the FGD reported that anthrax vaccination programs were irregular and mostly planned as a response to outbreaks, that is, there was no routine vaccination practiced.

## DISCUSSION

Here, we described three anthrax outbreaks that affected wildlife, livestock, and humans recurring in the same geographic location over a period of 4 years (2014–2017). In addition, PE identified and mapped prior anthrax outbreaks that occurred in the same locality in 1973, 1979, 1982, 1986, and 2011, suggesting that the region is a hotspot for *B. anthracis* persistence and anthrax outbreaks. Evidence from the Kenya Department of Veterinary Services disease surveillance records showed that between 2011 and 2016, Nakuru County reported the second highest number of livestock anthrax outbreaks in the country, with only Murang’a County in Central Kenya reporting more. In the outbreaks reported here, human cases of cutaneous, gastrointestinal, and oropharyngeal forms of anthrax were associated with contact and/or consumption of infected livestock as reported in other studies.^28–30^ No human cases were associated with the wildlife anthrax infections and death, likely because the LNNP is a protected area with patrolling rangers and perimeter electric fence to prevent human–wildlife interaction and conflict. Elsewhere in Kenya and Africa where wildlife exist in unprotected areas, there have been reports of human anthrax cases associated with slaughter and consumption of dead wildlife.^31,32^ Interestingly, most (46.7%) of the human cases in this study were gastrointestinal anthrax, including the two cases that died, contrary to outbreaks globally where cutaneous anthrax is the most common form.^2^ This is associated with the butchering dead animals and selling the meat cheaply or giving it free to neighboring households, a common practice among the poor, rural communities in sub-Saharan Africa.^3^ This finding is consistent with a WHO report indicating that in parts of Africa, India, and the southern Russia, one livestock anthrax case results in almost an equal number of gastrointestinal and cutaneous human anthrax cases.^2^

Although most households affected by the 2014 and 2017 outbreaks owned cattle, sheep, and goats, anthrax morbidity and mortality were only reported in cattle. Because the literature shows that all three livestock species are comparably susceptible to *B. anthracis* infection, the absence of cases in sheep and goats in Nakuru West subcounty may not be fully explained. However, review of anthrax outbreaks in Tanzania, Ghana, Bangladesh, and Italy showed higher incidence in cattle than in sheep or goats.^30,33–36^ A possible explanation is that the communities in the Nakuru West subcounty area practice communal grazing of cattle in neighboring uninhabited areas, thus exposing them to environments where *B. anthracis* spores are maintained. By contrast, sheep and goats are reared within the small-scale household farms where there is minimal exposure to ecosystems permissive to survival of the bacteria.

The 2015 outbreak was the largest wildlife anthrax outbreak documented in Kenya, resulting in death of more than 10% of the population of wildlife species affected. The African buffalo and the white rhino were the most affected reporting losses of > 15% of the park population. Grazing wildlife, including buffaloes and white rhinos had higher species-specific mortality rates when compared with browsers and other wildlife. These data indicate that anthrax outbreaks among wildlife can have devastating effects on wildlife conservation, resulting in significant reduction or complete loss of endangered animal species. Although the impact of epizootics on African wildlife has not been fully elucidated, the influence of emerging diseases, most of which are zoonotic, on species population dynamics has been reported.^36–39^ The difference in susceptibility between grazer and browser has been demonstrated in other outbreaks in Africa and could point to either differences in species susceptibility or behavioral vulnerability.^6,7,40^ Grazers feed closer to the soil where the *B. anthracis* spores persist, thus increasing the chance of ingesting or inhaling *the* spores.^7^ It is also possible that the shorter grass gets more heavily contaminated by infected carcasses.^17^

The recurrence of outbreaks in Baruti sub-location involving multiple species, including humans, livestock, and wildlife suggest that Nakuru West subcounty is an anthrax hotspot in the country. The outbreaks occurred during different seasons, with the 2015 and 2017 outbreaks occurring in July–August, which is at the end of the long rains and the coldest months in the country. By contrast, the 2014 outbreak occurred in February–March, which is at the end of the long dry season. Similar reports from other parts of the world indicate that anthrax outbreaks occur during both the hot-dry seasons before the rains, and also during the wet seasons.^7,41^ The PE findings identified eight more major outbreaks involved humans and livestock in the past 45 years. Despite reports indicating that Kenya experiences > 10 multi-species anthrax outbreaks every year, there have been no efforts to develop an occurrence or risk map that can help in the development of prevention and control strategies. Typically, the government responds to anthrax outbreaks by immediately deep-burying dead livestock and wildlife to reduce environmental contamination, ring vaccinating livestock, and treating affected humans. The presence of a risk map and an understanding of ecological risk factors can promote targeted livestock vaccination, public education, and early detection and response in wildlife, livestock, and humans.

The occurrence of the wildlife–livestock–human anthrax outbreaks may suggest that the meta-population is linked by mechanical dispersal of *Bacillus anthacis* by dogs, peri-domestic wildlife, and/or insects.^2,17,42^ A second and perhaps more likely hypothesis is that the local ecosystem supports and harbors persistent *B. anthacis*, and outbreaks that occur among diverse animal species, including wildlife and livestock, are independently transmitted and unlinked. However, involvement of humans is invariably associated with contact with or consumption of products from infected animals.^3^

This study had some limitations. We used Gram staining for *B. anthracis* as the confirmatory test even though this method has reduced specificity compared with other staining techniques.^28^ In addition, our definition of the number of people at risk as the total number of people found through active case searching and total number of people in households reporting at least one livestock case was an underrepresentation because there was exposures of community members living outside the affected households who assisted slaughtering or consumed meat from infected animals. Similarly, we used the total number of animals in households reporting at least one clinical case of anthrax as the population at risk. However, more animals from households that did not report animal cases in the area were likely exposed to anthrax because of the community grazing practices.

In summary, this study describes recurrent anthrax outbreaks over a 4-year period involving humans, livestock, and wildlife, and five prior outbreaks in the same locality in Kenya. Our follow-up studies are designed to identify ecological, demographic, and sociocultural factors associated with this and other hotspots in Kenya. The overall goal of the program is to develop an anthrax risk map for the country for use in developing prevention and control measures aimed at reducing the public health and economic impact of anthrax. Available interventions that the government may apply at these hotspots include routine livestock vaccination and community-specific public health education.
